# Benefits of Testosterone Hormone in the Human Body: A Systematic Review

**DOI:** 10.7759/cureus.78785

**Published:** 2025-02-09

**Authors:** Julio G Rojas-Zambrano, Augusto R Rojas-Zambrano, Andres F Rojas-Zambrano, Gabriela E Barahona-Cueva

**Affiliations:** 1 Gynecology, Dr. Regeneracion, Guayaquil, ECU; 2 General Physician, Dr. Regeneracion, Guayaquil, ECU; 3 General Physician, Universidad Central del Ecuador, Quito, ECU

**Keywords:** bone, depression, muscle strength, sexual function, testosterone, vascular endothelium

## Abstract

Testosterone is a key hormone with a complex and essential role in the physiology of healthy individuals; it is crucial for developing and maintaining muscle mass and improved bone density. In addition to these physical features, testosterone is vital for reproductive health as libido, erectile function, and spermatogenesis, the process of sperm production; its impact extends across multiple bodily systems, highlighting its importance for physical traits and overall health and fertility. This study aims to explore the critical and multifaceted role of testosterone in the physiology of healthy individuals. The method was to search PubMed from the year 1993 until current data using MESH terms: ((((testosterone) OR (androgens)) OR (testosterone insufficiency)) AND (healthy patients)) AND (testosterone replacement))). The inclusion criteria are studies with descriptive, observational, and experimental approaches on healthy patients that evaluated the action of testosterone. The updated review indicates that testosterone hormone supplementation positively influences several aspects, including sexual function, bone health, muscle strength, mood (particularly in reducing depression), and vascular endothelial function. However, these findings are limited by the small sample sizes and the relatively few studies available on this topic, warranting further research to better understand the full scope of testosterone's effects. Recent landmark trials have demonstrated that testosterone therapy offers modest benefits, particularly for older men with low testosterone levels and symptoms of hypogonadism. These benefits include improvements in sexual function, bone health, muscle strength, mood, and vascular endothelial function in healthy individuals. Given the potential benefits of testosterone therapy, ongoing research and clinical exploration are highly recommended to deepen our understanding of its full range of effects and to refine therapeutic strategies. Doing more studies will help clarify the role of testosterone in both healthy individuals and those with testosterone deficiency, leading to better-informed treatment approaches for the future.

## Introduction and background

Hormonal regulation is a cornerstone of human physiology, influencing a myriad of biological processes and maintaining homeostasis across various systems. Of the myriad hormones that orchestrate bodily functions, testosterone stands out due to its profound and multifaceted impact on health [1].

This introduction provides an overview of the significant hormonal changes that occur throughout the human lifespan, particularly emphasizing testosterone's essential role in maintaining various physiological functions. This systematic review explores testosterone's multifaceted functions, investigating its physiological significance, its regulatory mechanisms, and the potential consequences of its dysregulation.

Hormonal changes are intrinsic to human development and aging; beginning from fetal life to adulthood, the endocrine system, comprising glands such as the pituitary, thyroid, adrenal, and gonads, releases hormones that regulate growth, metabolism, reproduction, and mood [2]. These hormonal shifts are essential for normal physiological functions, but they also have implications for health and disease; in puberty, a surge in sex hormones, including testosterone and estrogen, drives the development of secondary sexual characteristics [3]. Testosterone, a steroid hormone primarily synthesized in the testes in males and in smaller quantities in the ovaries and adrenal glands in females, is essential for a wide range of physiological processes [3].

Testosterone operates through a feedback mechanism, which is crucial to understand. When testosterone levels rise, it leads to a decrease in the production of its primary source; on the other hand, when testosterone levels drop, it triggers an increase in its production; this feedback system is essential for understanding the regulatory mechanisms that govern this steroid hormone [4]. Several pathological conditions can impair the feedback mechanism between the hypothalamus and the gonads, highlighting the importance of accurately diagnosing the underlying cause to achieve optimal therapeutic outcomes. Reducing these hormones may result in diverse symptoms that can profoundly affect the patient's quality of life [5].

This systematic review aims to provide a comprehensive examination of the role of testosterone within the broader context of hormonal changes throughout the human lifecycle; identifying gaps in current research and exploring potential areas for future investigation are crucial for gaining a deeper understanding of testosterone’s role in health and disease.

## Review

Testosterone is a crucial steroid hormone primarily produced in the testes in males and, to a lesser extent, in the ovaries and adrenal glands in females. Its synthesis and regulation are highly complex processes that involve intricate interactions between multiple endocrine glands, including the hypothalamus, pituitary gland, and gonads, as well as feedback mechanisms that maintain homeostasis within the body [3].

In males, the majority of testosterone is synthesized in the Leydig cells of the testes, while in females, smaller amounts are produced by the ovaries and adrenal glands; testosterone is often considered a male hormone; it plays an essential role in both sexes, contributing to a range of physiological processes beyond sexual differentiation, including muscle mass maintenance, bone density, mood regulation, and metabolic function [3].

Testosterone production is governed by the hypothalamic-pituitary-gonadal (HPG) axis, a key endocrine system component involving dynamic interactions between the hypothalamus, pituitary gland, and gonads. The hypothalamus, located in the brain, initiates the hormonal cascade by secreting gonadotropin-releasing hormone (GnRH), which acts on the pituitary gland, stimulating the secretion of luteinizing hormone (LH) and follicle-stimulating hormone (FSH) [3].

In women, testosterone is produced in the ovaries, adrenal glands, and peripheral tissues. The ovaries produce testosterone from cholesterol, similar to that in males, involving conversion through various enzymatic pathways [3]. Testosterone regulates its production through negative feedback mechanisms; high testosterone levels inhibit the release of GnRH from the hypothalamus and LH from the pituitary gland, reducing further stimulation of testosterone production [2].

Testosterone is converted to dihydrotestosterone (DHT) by the enzyme 5α-reductase. DHT has a more potent androgenic effect and is crucial for the development of male secondary sexual characteristics and the maintenance of prostate health [2]. In males and females, testosterone can be aromatized to estrogen by the enzyme aromatase, influencing various physiological functions, including bone health and reproductive functions [3]. In general, in a healthy population, the normal level of testosterone hormone in men is an average of 264 to 916 ng/dL [6]. On the other hand, in women, the normal testosterone level is about 15-46 ng/dL [7].

Libido, or sexual desire, is the interest in engaging in sexual activities. Testosterone is critically involved in regulating libido in both men and women; it affects various brain regions involved in sexual desire, including the hypothalamus, which is responsible for sexual motivation and arousal [8]. Lower testosterone levels are associated with decreased vaginal lubrication; this reduction can lead to discomfort and pain during sexual activity, a condition often described as dyspareunia. Research indicates that testosterone therapy may improve vaginal lubrication and overall sexual satisfaction in women with low testosterone levels [9].

There is a huge association between testosterone level and bone density, which is related to a decrease in the testosterone hormone level, which will cause a reduction in bone density [10]. Testosterone is undoubtedly the primary testicular hormone affecting bone metabolism. It directly influences osteoclasts, osteoblasts, and osteocytes, facilitating periosteal bone formation during puberty and decreasing bone resorption throughout adulthood [11-12]. The surge in sex steroid production during puberty accelerates bone mineral accumulation and plays a role in creating sex-specific differences in bone growth. After mid-puberty, boys exhibit a greater increase in periosteal bone growth than girls, who, in turn, experience more significant endocortical bone formation [13].

Testosterone plays a crucial role in bone maturation at the end of puberty, helping bones achieve their peak mass, and is essential for maintaining bone density throughout adulthood. Testosterone also supports skeletal growth and homeostasis by enhancing mechanical loading [14]. Testosterone is a key regulator of muscle mass; it promotes protein synthesis and muscle hypertrophy by binding to androgen receptors in muscle cells. This anabolic effect enhances muscle growth and repair, making testosterone essential for maintaining muscle mass [15,16-17].

It is important to know that testosterone plays a significant role in muscle strength; it influences neuromuscular function, including muscle coordination and force production. Higher testosterone levels are associated with greater muscle strength and physical performance [15]. Research has shown that men with low testosterone levels experience reduced muscle mass and strength. Testosterone replacement therapy (TRT) can improve muscle mass, strength, and physical function in hypogonadal men [18]. Testosterone levels naturally decline with age, which can lead to sarcopenia (age-related muscle loss) and decreased muscle strength. Research has shown that testosterone therapy in older men can help counteract these effects, improving muscle mass and physical performance [19].

Testosterone's impact on mental health is multifaceted, involving a range of physiological and neurobiological mechanisms; also, medical research indicates that low testosterone levels are linked to symptoms of depression, and testosterone therapy has demonstrated the potential to improve depressive symptoms in some individuals [20]. It is crucial to consider that testosterone treatment may have antidepressant effects in patients with hypogonadism, including elderly individuals; evidence suggests that low testosterone levels are linked to depressive symptoms, and TRT could provide therapeutic benefits for those with testosterone deficiency [21].

Testosterone, a key steroid hormone, has been shown to significantly affect the vascular endothelium, the thin layer of cells lining blood vessels. This influence is important for maintaining vascular health and has implications for cardiovascular function [22]. The vascular endothelium regulates vascular tone, blood flow, and overall cardiovascular health. Testosterone supports endothelial function by enhancing nitric oxide production, promoting endothelial cell growth and repair, and reducing inflammation [22].

Materials and methods

**Figure 1:**
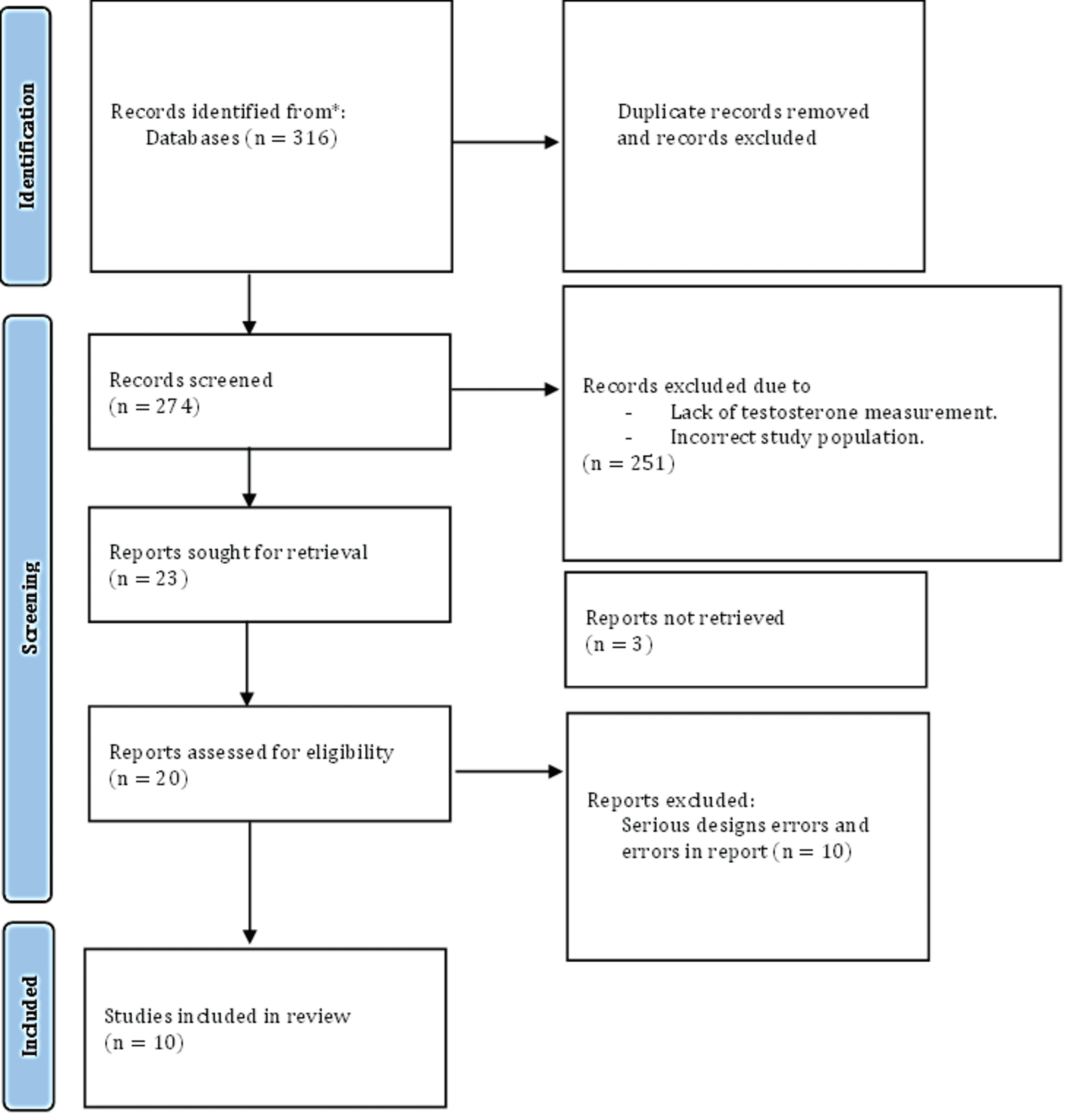
PRISMA flowchart PRISMA: Preferred Reporting Items for Systematic Reviews and Meta-Analyses

We performed a literature search and adhered to the guidelines specified in the Preferred Reporting Items for Systematic Reviews and Meta-Analyses (PRISMA) statement [23]. It is important to note that this research does not require ethical approval, as it involves a review of previously published articles related to patient data. A systematic search was done on PUBMED, and the following MESH term was applied: ((((testosterone) OR (androgens)) OR (testosterone insufficiency)) AND (healthy patients)) AND (testosterone replacement))). We used data from 1993 until the current articles. Publications that utilized descriptive, observational, or experimental designs involving human subjects and assessed the effects of testosterone were included based on the established criteria (Figure 1). The research encompasses testosterone's effects on sexual function, bone health, muscle strength, depression, and vascular endothelium (Figure 1) and presents a detailed overview of the selection criteria. To enhance understanding of the data, we present a table summarizing all collected information regarding comparing various aspects related to the ones mentioned before.

Results

Our study commenced with an initial collection of 316 articles, as shown in (Figure 1). After reviewing the titles and abstracts, we eliminated 42 articles due to duplication. Subsequently, during the full-text review, an additional 251 records were excluded; at the end of the review, 10 articles remained for further examination.

The systematic review included 10 studies (Table 1), all of which positively impacted various health outcomes, including sexual function, bone health, muscle strength, depression, and vascular endothelial function. These findings suggest significant benefits in these areas. However, conducting a well-designed randomized controlled trial (RCT) is essential to strengthen the evidence and provide more robust conclusions.

**Table 1:** Descriptive analysis of the studies RCTs: randomized controlled trials, TRT: testosterone replacement therapy, BMD: bone mineral density

Author(s), year	Study design	Aim of study	Setting and participants	Results and findings
Walther et al. (2019) [20]	Systematic Review and Meta-analysis	To examine the association of testosterone treatment with the alleviation of depressive symptoms in men and to clarify the moderating effects of testosterone status, depression status, age, treatment duration, and dosage	27 RCT	Testosterone treatment appears to be effective and efficacious in reducing depressive symptoms in men, particularly when higher-dosage regimens were applied in carefully selected samples
Cruickshank et al. (2024) [24]	Systematic review with meta-analysis	The main objective was to assess the safety of TRT	35 trials (5601 randomised participants)	TRT enhances sexual function and quality of life without impacting blood pressure, serum lipids, or glycemic markers
Xu et al. (2024) [25]	Systematic review with meta-analysis	Analyze the effects of TRT on erectile function and prostate	This analysis included and examined 28 RCTs with 3,461 patients	This meta-analysis of RCTs suggests that TRT may enhance erectile function in men with hypogonadism
Yang et al. (2023) [26]	Systematic review and meta-analysis	To conduct a systematic review and meta-analysis on whether TRT could improve sexual function in the elderly	5 RCTs	The improvement in both erection and motivation was clearly evident
Mauvais-Jarvis et al. (2024) [27]	Systematic review	This review discusses the metabolic benefits of estradiol and testosterone in both genders	157 references	Testosterone plays a crucial role in enhancing bone formation
Shigehara et al. (2021) [28]	Narrative review	The relationship between testosterone and BMD in men and mentioned the benefits of TRT on BMD among hypogonadal men	Articles published between January 1990 and October 2020 were gathered	TRT should be considered as one of the treatment options to improve hypogonadal symptoms and BMD simultaneously in symptomatic hypogonadal men with osteopenia
Green et al (2024) [29]	Systematic Review	To summarize what is currently known about the impact of T treatment, exercise, and their combination on body composition, strength, and aerobic fitness	A review of randomized studies to directly compare T treatment's combined and separate effects	Our review suggests that T has impacts on strength, body composition, and aerobic fitness outcomes that are dependent upon dose, route of administration, and formulation
Vartolomei et al. (2020) [30]	Systematic review	To investigate and critically evaluate the existing evidence on the effects of TRT on depression and depressive symptoms in adult men with late-onset testosterone deficiency in comparison to a placebo	1586 participants	According to data from small, placebo-controlled, randomized clinical trials involving patients with mild depression prior to treatment, TRT is associated with a reduction in depressive symptoms
Anderson et al (2022) [31]	Systematic review	The objective of this review is to synthesize information regarding clinical depression, its treatment options, and the efficacy and safety of testosterone treatment for the treatment of depression	This review employed an extensive analysis of secondary and tertiary data from various academic databases and published literature on the topic of interest	Testosterone administration yielded positive results in the treatment of depression
Kelly and Jones (2013) [32]	Narrative review	To assess the vascular hormone's role in health and disease	Review of 8 articles	Testosterone has shown clinically significant anti-inflammatory effects, and TRT can improve atherosclerosis, as evaluated non-invasively in hypogonadal men and animal studies

Such a study would offer a higher level of evidence, allowing for a more comprehensive understanding of the effects observed across the studies and helping to confirm the consistency and reliability of these results.

Risk of bias evaluation was conducted for each study, which is included in Table 2; two reviewers (JR and AR) independently evaluated potential biases across four critical domains: selection bias, measurement bias, confounding factors, and reporting bias. Each domain was rated as having a "low," "moderate," or "high" risk of bias; the other 2 authors (AR and GB) made the compilation and screening as well as reviewing the selected studies. The majority of studies exhibited a moderate risk of bias.

**Table 2:** Risk of bias assessment of included studies

Study	Selection bias	Measurement bias	Confounding	Reporting bias	Overall risk of bias
Walther et al. (2019) [20]	Low	Low	Low	Low	Low
Cruickshank et al. (2024) [24]	Moderate	Low	Moderate	Moderate	Moderate
Xu et al. (2024) [25]	Moderate	Low	Low	Low	Low
Yang et al. (2023) [26]	Moderate	Low	Moderate	Moderate	Moderate
Mauvais-Jarvis et al. (2024) [27]	Low	Moderate	Low	Low	Low
Shigehara et al. (2021) [28]	Low	Low	Moderate	Moderate	Low–Moderate
Green et al. (2024) [29]	Moderate	Moderate	Low	Moderate	Moderate
Vartolomei et al. (2020) [30]	Low	Low	Moderate	Moderate	Low - Moderate
Anderson et al. (2022) [31]	Moderate	Low	Moderate	Moderate	Moderate
Kelly and Jones et al. (2013) [32]	Low	Low	Moderate	Moderate	Low-Moderate

Discussion

Testosterone plays a crucial role in regulating libido in both men and women by influencing brain regions associated with sexual motivation and arousal [24]. Another research study points out that low testosterone levels are associated with low libido in 67.5% of patients, and the results are verified in that study by the following (OR 2.4; 95% CI: 1.2-4.8; p=0.01) [33].

Also, there is a notable link between testosterone levels and bone density. A decline in testosterone can lead to reduced bone density [27]. It is also important to understand the use of the Free Androgen Index (FAI), which is correlated with bone density; having a low FAI means that there is a higher probability of a decrease in bone density [34].

Additionally, testosterone is essential for muscle mass regulation, as it promotes protein synthesis and muscle hypertrophy by binding to androgen receptors in muscle cells [16]. The effects of testosterone treatment and exercise on variables like body composition, strength, and aerobic fitness significantly enhance our comprehension of the relative benefits of both physiological and pharmacological interventions for aging men [29].

In terms of mental health, testosterone may alleviate depressive symptoms in certain individuals. This is particularly relevant for patients with hypogonadism, including older adults, for whom TRT could provide significant therapeutic benefits [30,31].

Moreover, testosterone enhances endothelial function by promoting nitric oxide production, supporting the growth and repair of endothelial cells, and reducing inflammation. This function is critical for maintaining vascular health, highlighting testosterone's multifaceted role in overall physiological well-being [32]. Also, it is important to know that reduced testosterone levels are correlated with a higher incidence of atherosclerosis, coronary artery disease, and cardiovascular events, as well as decreased heart rate [35,36].

It is important to know that, as well as several medications having side effects, testosterone hormone is not the exception because it can cause hirsutism, the excessive growth of coarse, dark hair in male-pattern areas such as the face, chest, and abdomen. This occurs as testosterone stimulates androgen receptors in hair follicles, promoting increased hair growth. The severity of hirsutism is influenced by factors such as the dosage and form of testosterone (injections, patches, or gels), genetic predisposition to androgen sensitivity, and the duration of elevated testosterone levels, with individuals genetically predisposed to hyperandrogenism experiencing more pronounced hair growth. It requires careful consideration and management [37].

## Conclusions

Testosterone is a vital hormone that significantly influences various physiological processes throughout the human lifecycle. Its role extends beyond regulating libido in both men and women, as it is also closely associated with bone density and muscle growth and repair. At the same time, its impact on mental health, particularly in alleviating depressive symptoms, underscores its multifaceted nature. Furthermore, testosterone contributes to vascular health by enhancing endothelial function, which is crucial for cardiovascular performance.

This systematic review has provided a comprehensive examination of testosterone's physiological roles, emphasizing the need for further research to address existing gaps in knowledge. Continued exploration of testosterone's role may lead to improved therapeutic strategies that enhance the quality of life and health outcomes across diverse populations.
